# Understanding mental health conditions and key coping strategies utilized during major lockdowns in the Caribbean based on Google trends searches

**DOI:** 10.1016/j.heliyon.2023.e19843

**Published:** 2023-09-09

**Authors:** Richard Ramsawak, Preeya Mohan, Gerard Hutchinson

**Affiliations:** a The Business School, Royal Melbourne Institute of Technology – HCMC, Viet Nam; b Sir Arthur Lewis Institute of Social and Economic Studies, University of the West Indies, St Augustine Trinidad and Tobago; c Faculty of Medical Sciences, University of the West Indies, Mt Hope, Trinidad and Tobago

**Keywords:** COVID-19, Mental distress, Coping and English-speaking Caribbean

## Abstract

The COVID-19 pandemic has prompted countries to implement extended Shelter in Place Orders (SIPOs) to restrict population movement and mitigate community spread. While these lockdown measures may be effective in containing the virus, they can substantially impact the population's well-being, potentially undermining their overall welfare. This study investigates whether major lockdowns implemented in the Caribbean produced differential changes in mental health among key English-Speaking Caribbean countries. More importantly, unlike past studies, we examine key coping strategies persons utilize during major lockdowns. Finally, this paper utilizes a novel near real-time high-frequency data source in Google Trends data analytics to assess mental health patterns and coping strategies among major Caribbean countries. Based on the results of difference-in-difference and event study models, we find positive and significant increases in searches for fear, depression, and suicide during key lockdown periods, which suggest negative mental health effects. Regarding coping strategies, searches for Zoom, learning, books, exercise, prayer, religion, and meditation increased, together with searches for addiction and marijuana. These results indicate the types of programs health administrators and policymakers can implement during lockdown periods to help local mental health communities, particularly among island communities.

## Highlights

• This paper utilized data obtained from Google Trends to compare search frequencies of keywords related to mental health and coping strategies during the implementation of major COVID-19 lockdowns in the Caribbean.
• We compare search trends during major lockdown periods to the same period in the previous year and utilize difference-in-difference, event study, and regression discontinuity in design models to identify significant differences in search patterns.
• Our results suggest that implementing Stay in Place Orders (SIPOs) was associated with increased Google searches for anxiety, fear, depression, and suicide, which signal negative mental health effects. Additionally, searches for outdoor recreation and jobs and vacancies declined, reflecting the impact of SIPOs on restricting movement and economic activity. Regarding coping strategies, searches for learning, zoom, news, exercise, prayer, religion, and meditation increased, together with searches for addiction and marijuana.
• In terms of contribution, our research highlights the usefulness of near real-time big data sources, such as Google Trends analytics, in providing critical insight of mental health conditions among key Caribbean countries during a major health crisis such as COVID-19. This information is particularly relevant to Caribbean policymakers and decision-makers in an environment where access to timely and accurate data remains a challenge. Additionally, given extended lockdown restrictions placed on population movement during the height of the pandemic, Google Trends analytics is also used to determine coping strategies utilized by affected populations in the Caribbean. This is the second novel contribution of this paper to the literature, as it indicates the types of programs administrators and policymakers can implement to support the mental health of island communities during a crisis.

## 1 Introduction

In early 2020 the Coronavirus disease 2019 (COVID-19) emerged and affected almost every country, resulting in over 100 million confirmed infections and over 3 million deaths globally. The most common approach governments use to contain the spread of the virus has been Shelter in Place Orders (SIPOs) [[1], [2], [3], [4]]. SIPOs seek to limit transmission of COVID-19 by restricting population movement and social interaction so that the possibility of infections can be reduced, preventing national health services from being overwhelmed. SIPOs, while effective in reducing transmission of the virus, regrettably also have been found to impact economic activity negatively [5,6] It has also been associated with higher rates of business failure [5] and unemployment [6], increasing social isolation, and worsening mental health [7,8].

These events have spurred increasing research on the psychological outcomes of COVID-19 [[9], [10], [11]]. Research has shown a prevalence of psychological distress and depression [12], anxiety, and post-traumatic stress disorder, particularly among the vulnerable and those directly impacted by the virus, such as youth and front-line workers [13]. Higher levels of suicide, domestic violence, child abuse, and increasing use of alcohol and substance abuse are additional problems identified during the pandemic [14,15]. Past research found persons with specific affective temperament types (depressive, cyclothymic, hyperthymic, irritable, and anxious) prone to adverse clinical outcomes such as higher suicide behavior [16,17]. For these reasons, the impact of COVID-19 on mental health is becoming an increasing concern among policymakers and health practitioners, particularly in the face of multiple waves of infections and longer and more intensive SIPOs measures adopted by Governments [[18], [19], [20]]. Equally important but not fully explored in terms of research is understanding coping strategies (positive and negative) used by citizens to manage the social isolation and distress that come with prolonged lockdowns [21,22]. By understanding how people cope, new programs, and interventions can be designed and rolled out, which can be particularly useful for at-risk groups and communities during lockdown periods.

The study of the impact of COVID-19 on mental health is particularly relevant among Caribbean Small Island Developing States (SIDS). Island states share a common set of environmental, economic, and social vulnerabilities due to their relatively small size, geographical remoteness, and concentrated economic structures that make adjustments to external shocks such as COVID-19 difficult to mitigate [23,24]. Past research has shown that mental health disorders are a leading cause of disability and a major contributor to the burden of non-communicable diseases in the region [25,26]. Indeed, mental and neurological disorders account for 22.2% of the total burden of disease in the region (measured in disability-adjusted life years) [25]. The treatment gap for mental disorders in the Caribbean region is overwhelming and ranges from 37.4% (non-affective psychoses) to 64.0% (bipolar disorder)

Moreover, Caribbean countries have been found to be vulnerable to economic and climate shocks with severe mental health consequences [26]. In the past Caribbean countries have also had to deal with several epidemics, such as Dengue, Zika, and Chikungunya [27]. The Global Health Security Index (GHS Index) shows that small island economies have poorer health capabilities regarding prevention, detection, and rapid response to epidemics. The problem is also compounded by the high prevalence of non-communicable diseases such as diabetes, asthma, cardiovascular diseases, and obesity, which make people living in small island economies particularly vulnerable to developing severe symptoms from COVID-19 [23].

Caribbean countries have encountered notable disparities in infection rates, fatalities, and community transmission of COVID-19. 10.13039/100014337Furthermore, there is significant diversity among Caribbean governments in terms of the policy measures implemented to mitigate the spread of the virus and the level of support provided to impacted communities. Some island economies have adapted by creating “island bubbles,” becoming increasingly isolated from the mainland economies but simultaneously increasing interisland trade [28]. Additionally, Caribbean countries have had to rely largely on implementing varied SIPO measures, ranging from restrictions on the size of social gatherings, closure of schools, non-essential businesses, and curfews, frequently extending into months in some locations. These measures have significantly reduced community mobility within and across Caribbean countries and contributed to relatively low infection rates relative to comparator locations [23]. Despite the length and intensity of these measures, there remains little research focusing on the impact of COVID-19 on mental health conditions among Caribbean countries. Little research has also examined strategies people utilize to cope during major extended lockdowns. Like many emerging economies, Caribbean islands are plagued by a scarcity of timely data on key development issues, making policymaking during a crisis complex [[29], [30], [31], [32]]. This paper, therefore, seeks to examine the impact of major lockdowns on mental health among key English-speaking Caribbean SIDS.^1^ We do this by utilizing high-frequency near real-time data sources, based on internet search patterns during COVID-19 generated from Google Trends data analytics. The intuition of the approach is premised on the idea that the information-seeking behavior of individuals on the internet would reflect areas of interest, perception, and concern that are engaging their attention at the time of the search. Indeed, search patterns reported by Google Trends have been used to predict stock market trends [26,27] accurately, electoral outcomes [33,34], and tourism patterns [35]. Recent research has also linked internet search patterns to persons experiencing negative mental health crises in a given location. These people may increasingly search the internet to find information for support or end their suffering in the case of suicide [[36], [37], [38], [39]].

In terms of our approach, we apply several robust estimation techniques Difference-in-Difference (DiD), Dynamic Difference-in-Difference (DDD), and Regression Discontinuity Design (RDD) models to empirically assess differences in daily search patterns of key search terms related to mental health and coping both before and after the implementation of key lockdown measures among countries in the Caribbean. Unlike previous studies that utilize cross-sectional datasets, we compare search patterns during each country's main lockdown period to the same period in the prior year. In this regard, this paper closely follows Brodeur, Clark [40] and Knipe, Evans [22] by utilizing data gathered on keyword searches reported by Google Trends to assess mental health patterns among populations in key countries before and after the implementation of major lockdowns. However, we extend the mental health literature by examining strategies utilized by these communities to cope with SIPOs during COVID-19 lockdowns.

In terms of our contribution, to our knowledge, this is the first paper to seek to assess mental health and coping strategies utilized during COVID-19 in the English-speaking Caribbean. More importantly, access to timely, accurate, and reliable data to support policy development and decision-making remains a crucial challenge among Caribbean countries [23,41]. This issue becomes even more pressing during periods of national crisis. This paper aims to introduce a new alternative data source with supporting estimation techniques that can be used to robustly assess mental health conditions and coping strategies among key English-Speaking Caribbean countries during a national health crisis such as COVID-19. In this way, it is hoped that information can better support policy and key decision-makers in providing social and community support during similar health and national crises in the future.

The remainder of this paper is structured as follows; Section 2 provides the data and methodology used for the study. Section 3 outlines the results, while section 4 provides concluding remarks.

## 2 Data and methodology

### 2.1 Data

Data for this study were drawn primarily from the Google Trends dataset. Google Trends is a public website that offers data based on the frequency with which a particular search term is used on the Google Inc search platform. Search queries for words are defined in either terms or topics. Queries based on search terms include matches for an exact word or phrase made in the Google search box. Topics searches are broader and comprise related terms that share the same concept in any language.^2^ Ginsberg, Mohebbi [42] pioneered its use by detecting the spread of influenza in the USA before the Centre for Disease Control using data from the Google search engine. Similar to previous research employing Google Trends analytics, we assume that search indicators offer reliable and indicative information about the current behaviors and sentiments of Google Search users [[43], [44], [45]]. Indeed, recent research has attempted to link Google Trends search patterns to heightened suicide risk in the case of Italy [39].

Data on search patterns continue to be freely accessible through Google Trends web portals. Google Trends returns daily search results for exact keywords relative to the total number of all Google topics over a given period for a specific geographic area. Google Trends also normalizes search data per search locale by generating a relative score between 0 and 100 across the period selected. A value of zero represents the minimum level of search popularity in the specified frame, indicating that a search term did not have a search volume on that given day. While 100 represents the maximum level of search popularity. The availability of daily search data is limited to a query period of less than nine months, and it is provided up to 36 hours before the search requests are made. For search queries over nine months and up to five years, weekly rather than daily data are provided.^3^

We collect data for key English-speaking CARICOM SIDS, Caribbean countries with the largest populations and high internet/cell phone penetration rates and usage that implemented full lockdowns during the survey period considered. These countries include the Bahamas, Barbados, Guyana, Jamaica, and Trinidad and Tobago (see Table 1A, Table 2A). We collected data for the period 1st January and September 1, 2020 and the same period in 2019, which is used as our base period. Since the daily data score for 2019 searches is obtained through a distinct request from the daily data in 2020, the indexed scores cannot be compared due to this differentiation. We utilized a scaling procedure developed by Brodeur, Clark [40] to create a rescaled index that can be used to compare search trends of keywords over the sample period (see Appendix).

### 2.2 Selection of key search terms

Our selection of key search terms was based on questions typically used to assess the quality of mental health and key coping strategies used during crisis events [21,40,46]. For instance, to ascertain interest in mental health, we derive keywords from the Kessler Psychological Distress Scale (K10) Survey [47] an instrument used to assess mental health and stress patterns during COVID-19 [[48], [49], [50], [51]]. Specifically, we examined searches for keywords such as anxiety, fear, suicide, and depression. Regarding coping, this can be defined generally as the conscious or unconscious cognitive and behavioral strategies individuals utilize to manage stress [52,53]. Coping strategies are varied, as are the underlying theoretical frameworks which attempt to explain it [54,55]. For this study, we utilized the conceptual framework developed by Carver, Scheier [56] and operationalized in the *Brief COPE* survey [57]. Based on this, coping strategies can be classified as problem-solving or active coping, self-distraction, religious coping, self-blame or self-abuse, and seeking social support. We also follow Knipe, Evans [22] by selecting terms based on the World Health guidelines for coping and resilience during COVID-19 [58]. The choice of search terms for all categories was also customized to address Caribbean language and cultural peculiarities. However, we focused on words which would be commonly interpreted across locations. Additionally, as a further step to quality-check these results, for each word utilized in this study, we closely examined Related Topics and Related Queries to ensure a consistent interpretation across locations (see Appendix Fig. 1A - Sample of Related Topics and Related Queries for a keyword search for “Anxiety” in Trinidad and Tobago over the period January 1, 2019 to 2020). Finally, we excluded topics with low to moderate *relative search volumes* (RSVs)^4^ and were stable over time. Based on this we expect that as lockdown periods intensify, Google searches for the final list of keywords to increase over time (Table 1).

**Table 1:** Topics included in keyword searches.

Topic	Key search term
Mental Health	Anxiety
	Fear
	Suicide
	Depression
Active coping	Beach
	Exercise
	Learning
	Books
	Jobs
Religious coping	Meditation
	Prayer
	Religion
Avoidance, distracting coping	Movies
	Music
	Games
Seeking social support and networking	Facebook
	Instagram
	Twitter
Abuse coping type	Abuse
	Violence
	Marijuana
	Porn
	Addiction

### 2.3 Dates of key lockdown periods

We also utilized information from the COVID-19 Response Tracker to understand differences in Government response to country-level changes in COVID-19 infections over time (see Appendix Fig. 2A). Country-level data on population levels were also used to weight Google search trends. Finally, information on daily COVID-19 infections was obtained from Johns Hopkins COVID-19. While country-specific data such as population levels were gathered from the World Bank Development Indicators database.

### 2.4 Methodology and data analysis

#### 2.4.1 Difference-in-difference models

Our identification strategy is based on empirically estimating differences in search patterns of keywords related to mental health and coping during major lockdown periods among key Caribbean countries relative to search patterns of the same words during the same period in the previous year before the emergence of the COVID-19 virus. To this end, we utilized difference-in-difference models to assess these changes in magnitude accurately. We closely follow the work of Brodeur, Clark [40] by comparing searches pre-and-post lockdown after major lockdowns were implemented in 2020, to a comparative period in 2019. We, therefore, estimate our first model based on equation (1).

(1) $$Y_{it}=\alpha T_{it}*{Year}_{t}+{\beta T}_{it}+{\lambda Cases}_{it-1}+p_{i}+u_{t}++\epsilon_{it}$$

In this case $Y_{it}$ represents the number of searchers for a specific topic, and α **i**ndicates the impact of the lockdown on Google searches related to that topic. T represents a dummy variable taking a value of one in the days after the main lockdown period starts and zero otherwise. The year of the lockdown is given by ${Year}_{t}$ which in this case is 2020. The number of confirmed COVID-19 cases in each country *i* is given by Cases***.*** The model incorporates country fixed effects, as well as fixed effects for year, week, and each day of the week (from Monday to Sunday), denoted as $u_{t}\text{.}$ The standard errors are estimated using a robust approach and clustered at the day level.

Our identification strategy leverages differences in lockdown periods across Caribbean countries to the extent that this can influence differences in search patterns across individual countries. The key assumption is that in the absence of the lockdown, Google users’ behaviors would have evolved the same way as in the year before the lockdown.

#### 2.4.2 Dynamic Difference-in-difference models

We also utilize Dynamic Difference-in-Difference (DDD) and Regression Discontinuity in Design (RDD) models as a robustness check. Specifically, we examine the impact of lockdown measures on search patterns up to four weeks leading up to and five weeks after the implementation date of major lockdowns relative to the same weeks in the previous year. To do this, we estimate our DDD model based on equation (2)

(2) $$Y_{it}={\sum_{k=-4}^{k=5}\alpha_{k}W}_{ki}*{Year}_{t}+\sum_{k=4}^{k=5}\theta_{k}W_{ki}+{\lambda Cases}_{it-1}+p_{i}+u_{t}++\epsilon_{it}$$

Our coefficient of interest is $\alpha_{k}$ examines the impact on keyword searches after each week ***k*** both before and after the lockdown period. The estimated effect is relative to the same week in the previous year. Once again, we include controls for the number of new cases (lagged by one day), with country, week, and day-of-week fixed effects. Standard errors continue to be clustered at the day level.

#### 2.4.3 Regression discontinuity design

Finally, an alternative approach will be to utilize the Regression Discontinuity Design (RDD) methodology. Specifically, we examine the extent that structural breaks exist in two non-parametric series, one modeled five days before and the second five days after the major lockdown period in 2020. We also compare the extent to which structural breaks exist over the same period in2019.^5^ The collective results of these models will be used to formally test the impact of implementing major lockdowns on search patterns.

## 3 Results

### 3.1 Descriptive analysis

We begin by comparing fundamental trends in the frequency of key search terms related to mental health and key coping strategies during major lockdown periods. To conduct our analysis, we specifically examine search trends for keywords during the corresponding periods of 2020 and 2019. Fig. 1 illustrates the daily search activity before and after the lockdown in 2020 compared to the same period in 2019. The graph focuses on a selection of keywords associated with mental health and coping, capturing the changes during the pre- and post-lockdown periods in 2020 relative to the corresponding time frame in 2019. In the sample charts, we observe a significant rise in searches for anxiety and depression immediately following the implementation of nationwide lockdowns in 2020. We also observe positive spikes up to 60 and 70 days after lockdown protocols were instituted, reflecting the renewed interest in these search terms well into the lockdown period. Regarding positive coping strategies, we noted a marked increase in searches for learning, while searches for beaches and jobs were declining in 2020 relative to 2019, reflecting the impact of lockdown measures and softening economic activity during the COVID-19 period in 2020. Finally, we found a moderate increase in searches for negative coping strategies such as marijuana between 2020 and 2019 pre- and post-lockdown trends. We, therefore, turn to difference-in-difference models to empirically estimate differences in search patterns.

**Fig. 1:**
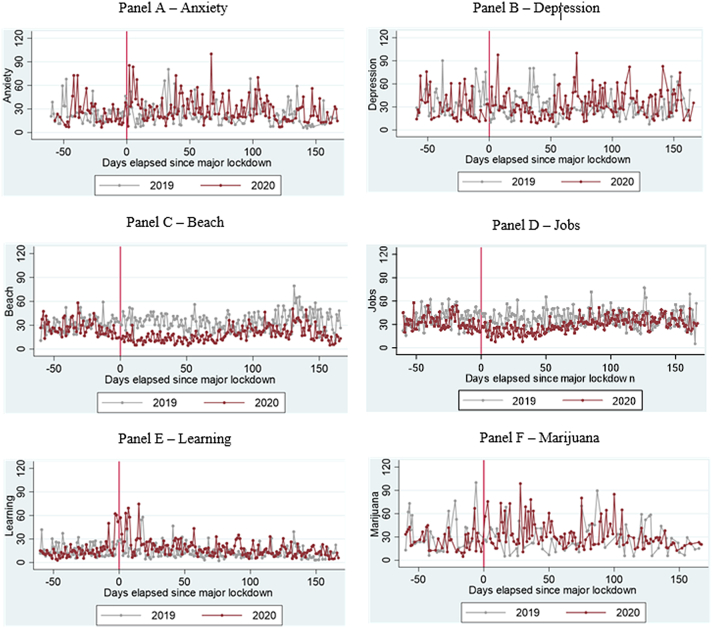
**Google Trends search patterns related to a sample of keywords associated with the quality of mental health and coping strategies before and after implementing major Shelter in Place Orders (SIPOs) among key Caribbean countries (Panel A – Anxiety, Panel B – Depression, Panel C – Beach, Panel D – Jobs, Panel E - Learning, and Panel F – Marijuana).** On the vertical axis, the index scores of searches are displayed for the days preceding (indicated by negative values) and following (indicated by positive values) the announcement of Shelter in Place Orders (SIPOs) in 2020. The red dots represent the index scores for 2020, while the grey dots represent the index scores for the corresponding period in 2019. The dots represent the raw averages calculated for each day, grouped into bins of one day, and are weighted by the population levels of each country. The countries included in the analysis are the Bahamas, Barbados, Guyana, Jamaica, and Trinidad and Tobago.

### 3.2 Results of difference-in-difference estimates

Table 2, Panels A to E, displays the results of difference-in-difference estimates, allowing us to evaluate the extent of lockdown measures' impact on search patterns in 2020 compared to the equivalent period in 2019. Firstly, results for mental health are provided in Table 2 Panel A, where we find a positive and significant increase in searches for fear, depression, and suicide after the lockdown period in 2020 relative to the same period in 2019.

**Table 2:** **Panels A and B display the results of DiD estimates**. The model incorporates controls for country, year, and day-of-week fixed effects and lagged values of new infections per 100,000 people. The estimates are weighted by population size, and the standard errors are clustered at the day level. **Panels C, D, and E display the results of DiD estimates**. The model incorporates controls for country, year, and day-of-week fixed effects and lagged values of new infections per 100,000 people. The estimates are weighted by population size, and the standard errors are clustered at the day level. *p < 0.10 **p < 0.05 ***p < 0.01.

Panel A				
	Anxiety	Depression	Fear	Suicide
Post lockdown*Year	18.62	26.89**	21.62**	20.27***
	(16.51)	(11.02)	(9.78)	(6.22)
N	370	344	319	202
R-sq	0.195	0.183	0.249	0.325
adj. R-sq	0.089	0.066	0.132	0.141

Panel B						
	Beach	Exercise	Books	Learning	News	Jobs
Post lockdown*Year	−1.73	10.21**	10.72***	15.30*	−3.90	0.55
	(3.53)	(4.69)	(4.09)	(8.78)	(3.27)	(3.32)
N	818	534	697	578	1109	830
R-sq	0.260	0.218	0.110	0.293	0.436	0.361
adj. R-sq	0.219	0.149	0.051	0.236	0.413	0.327

Panel C			
	Meditation	Prayer	Religion
Post lockdown*Year	32.57***	4.25	12.38*
	(12.09)	(5.78)	(7.00)
N	122	749	261
R-sq	0.522	0.121	0.438
adj. R-sq	0.294	0.066	0.324

Panel D						
	Movies	Music	Games	Facebook	Instagram	Twitter
Post lockdown*Year	5.83	−3.36	1.45	−2.66	2.55	−2.47
	(3.88)	(4.03)	(4.29)	(3.35)	(5.39)	(8.01)
N	1098	1025	1046	1109	1084	838
R-sq	0.442	0.163	0.130	0.334	0.383	0.108
adj. R-sq	0.419	0.127	0.092	0.307	0.358	0.060

Panel E					
	Abuse	Violence	Marijuana	Porn	Addiction
Post lockdown*Year	2.75	1.18	22.29**	−4.91	16.54***
	(5.01)	(9.90)	(9.76)	(3.37)	(5.28)
N	239	172	230	1110	90
R-sq	0.384	0.566	0.235	0.339	0.805
adj. R-sq	0.248	0.424	0.058	0.312	0.684

Regarding positive coping strategies (Table 2 Panel B), we find a positive and significant increase in active coping searches related to exercise, books, and learning. We also find a positive and significant increase in religious coping strategies, specifically for searches pertaining to religion and meditation (Table 2 Panel C). Interestingly, we find no significant increases in searches for keywords related to distractive coping strategies (Movies, Music, and Games). This result appears to be contradictory to global patterns, which highlight excessive technology and internet use, particularly in the streaming of movies, music, and online gaming during lockdowns (see Sharma, Anand [59] and Huang, Chen [60]). Also interesting to note is the limited growth in searches for online social networking apps such as Facebook, Twitter, or Instagram during the lockdown period (Table Panel D). Finally, Table 2 Panel E provides difference-in-difference results for negative coping strategies, which shows that searches for addiction and marijuana are positive and significant.

## 4 Results of robustness tests

As a robustness test, we examine alternative dates, such as when partial lockdowns were implemented as well as reopening dates, and find that these results are largely consistent with when major lockdowns were implemented. Additionally, based on the results of our DDD models, we confirm significant increases in searches for depression, mainly in the weeks after the implementation of major lockdowns (see Fig. 2 – Panel B). Our results also confirm key active coping strategies such as exercise and learning (see Fig. 3 – Panels B and C) and religious coping strategies, specifically in searches for religion during the survey period relative to 2019 (see Fig. 4 – Panel A). We find marginal increases in searches for Games and Facebook in the weeks leading up to and after the implementation of major lockdowns (see Fig. 5 – Panels C and D), reflecting the week-by-week changes in relative search patterns. Finally, we confirm increased searches for marijuana and addiction weeks after major lockdowns were implemented (see Fig. 6 Panels C and E).

**Fig. 2:**
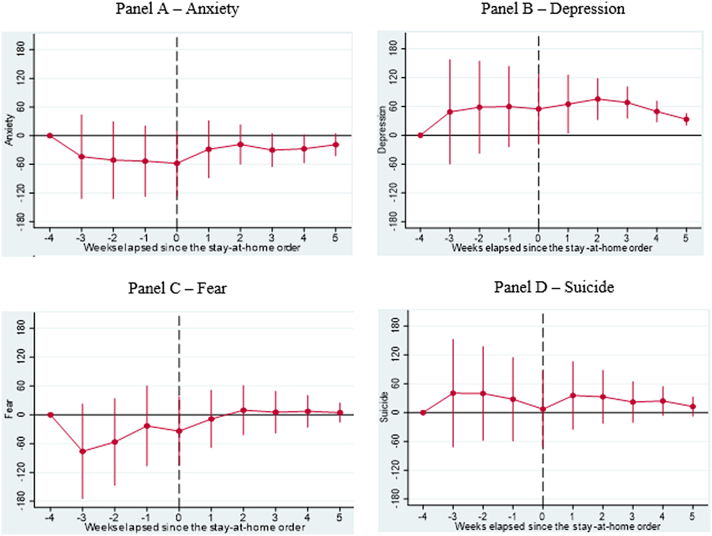
**Results of Dynamic Difference in Difference (DDD) Models based on** Google Trends **searches related to mental health generated b**efore and after implementing **Shelter in Place Orders (SIPOs)** among Caribbean Countries (Panel A – Anxiety, Panel B – Depression, Panel C – Fear, and Panel D – Suicide). The vertical axis represents the outcomes of DDD models, which offer estimates of the variations in search patterns for specific keywords during week *k* in 2020, compared to the corresponding period in 2019, both before and after the implementation of significant lockdown measures. These models account for the country, year, week, and day-of-week fixed effects and the lagged number of new infections as control variables. Population weights are applied to the analysis, and the standard errors are clustered at the day level.

**Fig. 3:**
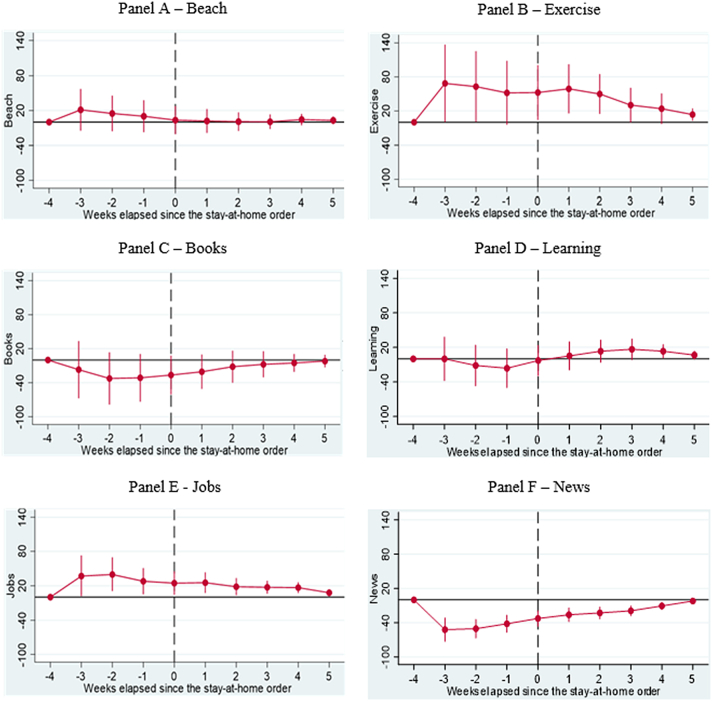
**Results of Dynamic Difference in Difference (DDD) Models based on** Google Trends **searches related to key positive coping strategies generated b**efore and after implementing**Shelter in Place Orders (SIPOs)** among key Caribbean Countries (Panel A – Beaches, Panel B – Exercise, Panel C – Books, Panel D – Learning, Panel E − Jobs, and Panel F – News). The vertical axis represents the outcomes of DDD models, which offer estimates of the variations in search patterns for specific keywords during week *k* in 2020, compared to the corresponding period in 2019, both before and after the implementation of significant lockdown measures. These models account for the country, year, week, and day-of-week fixed effects and the lagged number of new infections as control variables. Population weights are applied to the analysis, and the standard errors are clustered at the day level.

**Fig. 4:**
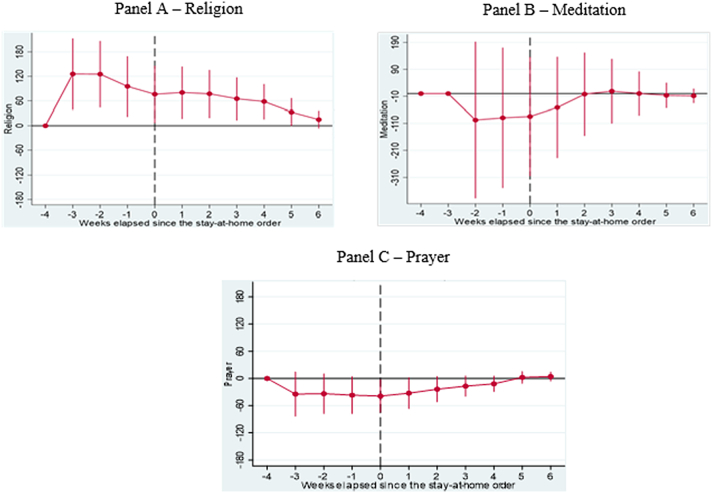
**Results of Dynamic Difference in Difference (DDD) Models based on** Google Trends **searches related to key religious coping strategies generated b**efore and after implementing **Shelter in Place Orders (SIPOs)** among key Caribbean Countries (Panel A – Religion, Panel B – Meditation, and Panel C – Prayer). The vertical axis represents the outcomes of DDD models, which offer estimates of the variations in search patterns for specific keywords during week *k* in 2020, compared to the corresponding period in 2019, both before and after the implementation of significant lockdown measures. These models account for the country, year, week, and day-of-week fixed effects and the lagged number of new infections as control variables. Population weights are applied to the analysis, and the standard errors are clustered at the day level.

**Fig. 5:**
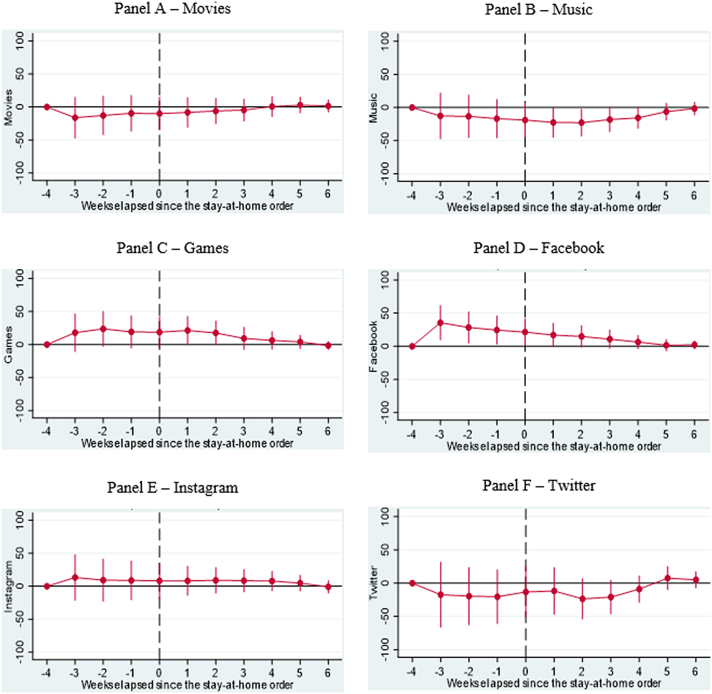
**Results of Dynamic Difference in Difference (DDD) Models based on** Google Trends **searches related to key distractive coping strategies generated b**efore and after implementing **Shelter in Place Orders (SIPOs)** among key Caribbean Countries (Panel 5A – Movies, Panel 5B – Music, Panel 5C – Games, Panel 5D – Facebook, Panel 5E – Instagram, and Panel 5F – Twitter). The vertical axis represents the outcomes of DDD models, which offer estimates of the variations in search patterns for specific keywords during week *k* in 2020, compared to the corresponding period in 2019, both before and after the implementation of significant lockdown measures. These models account for the country, year, week, and day-of-week fixed effects and the lagged number of new infections as control variables. Population weights are applied to the analysis, and the standard errors are clustered at the day level.

**Fig. 6:**
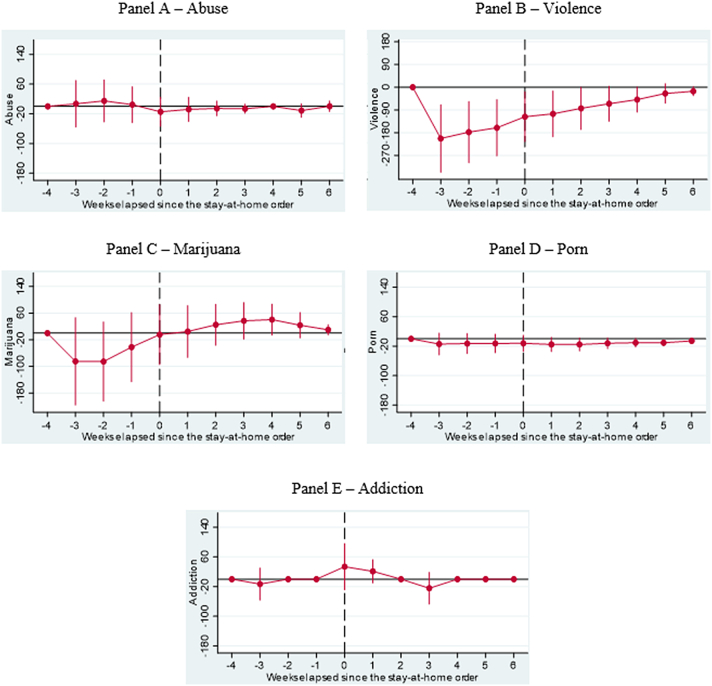
**Results of Dynamic Difference in Difference (DDD) Models based on** Google Trends **searches related to key negative coping strategies generated b**efore and after implementing **Shelter in Place Orders (SIPOs)** among key Caribbean Countries (Panel 6A – Abuse, Panel 6B – Violence, Panel 6C – Marjunia, Panel 6D – Porn and Panel 6E – Addiction). The vertical axis represents the outcomes of DDD models, which offer estimates of the variations in search patterns for specific keywords during week *k* in 2020, compared to the corresponding period in 2019, both before and after the implementation of significant lockdown measures. These models account for the country, year, week, and day-of-week fixed effects and the lagged number of new infections as control variables. Population weights are applied to the analysis, and the standard errors are clustered at the day level.

**Fig. 7A:**
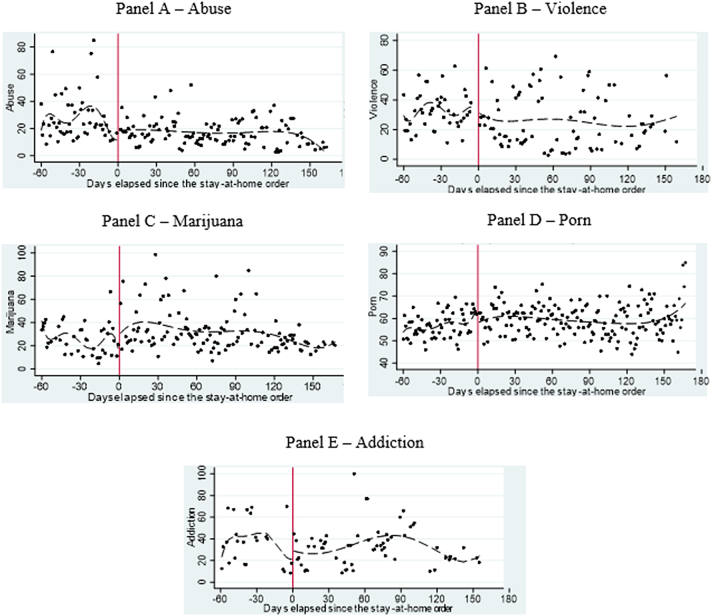
Results of RDD (2020) models based on research patterns for keywords related to negative coping strategies generated before and after implementing **Shelter in Place Orders (SIPOs)** among key Caribbean Countries. (Panel A – Abuse, Panel B – Violence, Panel C – Marijuana, Panel D – Porn, and Panel E − Addiction). - On the vertical axis, the scaled number of searches (ranging from 0 to 100) is displayed for the days preceding (represented by negative values) and following (represented by positive values) the announcement of the Shelter-In_Place Orders (SIPOs) in 2020. TheShelter in Place Orders announcement is considered day zero. The dots on the graph represent the raw averages calculated for each day, grouped into bins of one day for five countries, weighted by the respective population size of each country.

Finally, RDD models confirm a statistically significant and positive increase in searches for depression and fear following the implementation of major lockdown measures. (see Fig. 3A). We also find largely positive breaks in searches for positive active coping strategies, precisely exercise, learning, and books. Interestingly searches for news, while high leading up to the implementation of the major lockdown, declined significantly during the post-lockdown period (see Fig. 4A). There is also evidence of positive breaks in search patterns for meditation, and prayer after implementing lockdown protocols, providing further evidence of the importance of religious coping strategies (see Fig. 5A). The RDD for the year 2020 largely supports the results of the difference-in-difference models, which showed limited changes in searches for music and key social media handles between the pre and post-lockdown period (see Fig. 6A). While notable positive and significant differences were estimated in pre and post-lockdown searches for marijuana and addiction during the 2020 lockdown period (see Fig. 7A).^6^

## 5 Discussion

Overall our results confirm a positive and significant increase in searches for depression, fear, anxiety, and suicide after implementing major lockdowns among key Caribbean countries. An effect similar to Google search patterns found in other locations, such as the United Kingdom, the United States, and Latin America during the pandemic [22,40,61]. Indeed, past studies by Caribbean P.A.H.O [26] also found a heightened sense of fear in the Caribbean from previous disease outbreaks, such as Cholera and pandemic influenza, driven by the invisibility of the agent and uncertainty about contagion and risk of future infections.

Regarding key positive coping strategies utilized by communities during lockdowns, we find an increase in searches for learning, books, exercise, religion, and meditation relative to the same period in the previous year, reflecting the relative importance of active and religious coping strategies. This result is consistent with previous research, highlighting the importance of physical exercise and religion in maintaining positive mental health during the pandemic [21,62,63]. Additionally, for the Caribbean region, in particular, religion continues to play an essential role in the lives of communities as religious institutions have been known to provide education, sustenance, and shelter during times of crisis [26].

Interestingly, unlike more developed country contexts, we find limited growth in searches for key social networking sites such as Facebook and Twitter during the implementation of major lockdowns. One explanation can be that people may log in to these sites directly using phone or computer apps rather than through Google searches. However, it may also reflect the limited increase in new users, which would have been expected during major lockdown periods. These results may also be evidence of the continuing challenges of connectivity and digital integration among specific groups across Caribbean countries [64,65]. Finally, we find evidence of a rise in searches related to addiction and marijuana, suggesting a notable increase in these negative coping strategies during extended lockdowns. This result is consistent with past evidence of excessive alcohol and drug consumption in the region during past crisis events [26]. More worrying, these negative coping strategies can lead to a further decline in individuals' mental health and quality of life during the post-lockdown period. For instance, Budimir, Probst [21], find alcohol and cigarette consumption to be negative predictors of psychological life quality and well-being and positive predictors of perceived stress, depression, anxiety, and insomnia, up to four weeks after lockdowns were initiated in the case of Austria**.**

Collectively, these results provide significant evidence of the usefulness of new high-frequency big data sources to researchers and policymakers during periods of crisis, particularly in locations where reliable and timely data may be challenging to obtain. Additionally, this research examines how Caribbean island populations responded to and coped during the implementation of the extended lockdowns of COVID-19. Policymakers and health officials can utilize this information to enhance their strategies and better support and protect communities during similar crises. For instance, policymakers can prioritize initiatives that promote awareness and availability of books and online learning resources. By leveraging smartphones and other digital devices, educational materials can be made more accessible to individuals. This can also help mitigate the negative impact of disrupted education and support continuous learning. Policymakers can also collaborate with relevant organizations and promote online exercise, meditation, and self-help programs. Recognizing the vital role religious institutions play in supporting and guiding communities in the Caribbean. Policymakers can collaborate with these institutions, leveraging their existing networks and trust to disseminate accurate information, provide emotional support, and offer resources to affected communities during crises. Finally, Governments can implement policies to discourage negative coping behaviors such as self-abuse, self-harm, and addiction. These policies can include awareness campaigns, educational programs, and legal measures that promote healthy coping mechanisms and provide information on available support services. Recognizing the potential long-term consequences of addictive behavior, policymakers can allocate resources and support for rehabilitation and recovery programs. These programs can help individuals recover from the adverse effects of the pandemic, including mental health issues and substance abuse, and promote overall well-being in the post-pandemic period.

### 5.1 Strengths and limitations of the study

Our study utilizes high frequency near real-time data generated by Google Trends searches to assess mental health conditions and identify possible coping strategies of Caribbean communities during national lockdowns. Since Google search data provides aggregate measures of search activity at the country level, it is less prone to exhibiting small-sample bias [66]. Moreover, it remains unaffected by survey data biases, such as the observer-expectation effect or interviewer bias. Additionally, we use doubly robust methods in DiD and DDD models to test the reliability of these results.

Despite the real-time informational advantages of Google Trends Analytics, some limitations remain. For instance, younger individuals are more likely to use Google Search than older individuals. Additionally, the available data does not enable us to examine the heterogeneous effects of lockdown measures across different demographic groups, such as age, gender, or income class. This limitation is especially relevant for vulnerable populations, who may employ distinct coping strategies compared to the general population. Ideally, this approach can be combined with new survey methods analysis, such as high-frequency phone surveys [67,68], better to support policy and decision-makers during periods of crisis.

## 6 Conclusion

COVID-19 has posed significant mental health challenges to populations worldwide. However, there is a notable lack of studies investigating the mental health impacts and coping strategies employed by affected populations in the Caribbean Small Island Developing States (SIDS) during the pandemic. It is crucial to address this research gap to understand the mental health effects of COVID-19 and identify effective coping measures. Such knowledge is essential for managing the pandemic more effectively and preparing for its future consequences. Furthermore, the availability of timely data remains a crucial challenge for policymakers and decision-makers in the Caribbean. This study aims to fill the research gap while simultaneously addressing this issue. Specifically, we utilize a unique near real-time data source derived from Google Trends searches and employ doubly robust DiD and DDD models to access the mental health impact and coping strategies used by communities in key Caribbean SIDS during major COVID-19 lockdowns.

Our findings confirm negative mental health consequences of implementing major extended lockdowns among key Caribbean SIDS during the COVID-19 pandemic. Additionally, our results indicate that individuals employed active and religious coping strategies to positively navigate the lockdowns, as evidenced by increased searches for books, learning, exercise, religion, and meditation. However, there is also evidence of negative coping strategies, reflected in the increased searches for addiction and marijuana.

These results have important policy implications. They suggest renewed efforts to raise awareness and improve the availability of online learning resources and self-help programs within affected communities. Additionally, there is an opportunity to leverage the support and outreach provided by religious groups and denominations during the COVID-19 pandemic. Governments can also enact policies to discourage negative coping behaviors such as self-abuse, self-harm, and addiction, as these behaviors can lead to further problems beyond the pandemic.

The insights gained from this study highlight the usefulness of new data sources in assessing mental health conditions during major health crises and their potential application to other disaster-related events. However, it is essential to acknowledge the presence of certain limitations. For instance, while Google Trends data provides valuable insights, it does not allow for a granular examination of mental health or coping strategies among specific demographic groups. This includes low-income earners, individuals with pre-existing mental health issues, older persons with comorbidities, and healthcare workers. Furthermore, there is a generational disparity in the use of the Google Search engine, with younger individuals being more inclined to utilize it due to their familiarity and ease of internet usage. As a result, there is an opportunity for further research in this field, potentially combining the outcomes of primary surveys with Google Trends data queries to obtain a more comprehensive understanding of mental health and coping strategies across diverse demographic groups.

## Author contribution statement

Richard Ramsawak: Conceived and designed the experiments; Performed the experiments; Analyzed and interpreted the data; Contributed reagents, materials, analysis tools or data; Wrote the paper.

Preeya Mohan, Conceived and designed the experiments; Performed the experiments; Analyzed and interpreted the data; Contributed reagents, materials, analysis tools or data; Wrote the paper.

Gerard Hutchinson: Conceived and designed the experiments; Analyzed and interpreted the data; Wrote the paper.

## Data availability statement

Data is available on request.

## Declaration of competing interest

The authors declare that they have no known competing financial interests or personal relationships that could have appeared to influence the work reported in this paper.

## Figures and Tables

**Fig. 1A:**
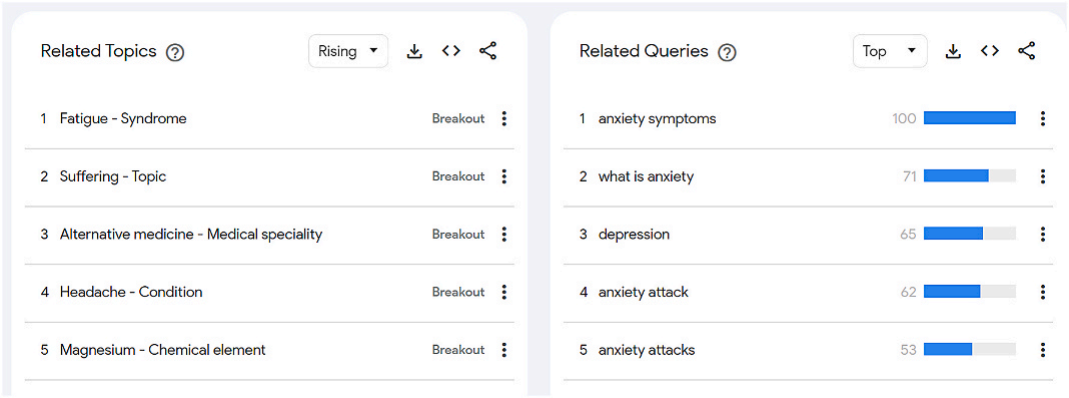
Related Topics and Related Queries based on Google Trends keyword search of“Anxiety” for Trinidad and Tobago January 1, 2019 to December 31, 2020.

**Fig. 2A:**
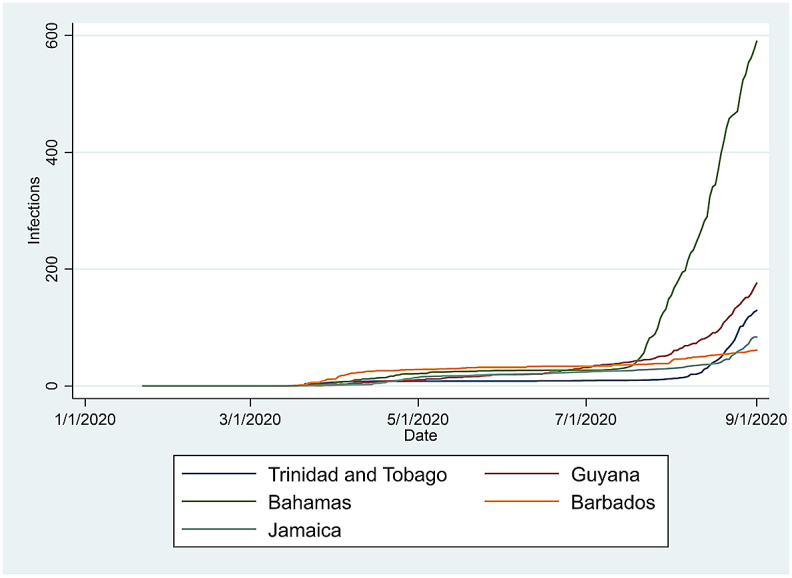
COVID-19 Infection rates among key Caribbean countries. Source: Johns Hopkins University COVID-19 Tracker

**Table 1A:** Internet penetration rates and usage among key Caribbean countries in 2020.

Country	Population (2021 Est.)	% Penetration	GDP per capita US$PPP (2019)
**Bahamas**	**399,285**	**83.4**	**38,742.5**
**Barbados**	**286,388**	**79.9**	**16,330.9**
**Trinidad and Tobago**	**1,372,598**	**77.3**	**27,334.2**
**Jamaica**	**2,898,677**	**54.5**	**10,193.5**
**Guyana**	**782,225**	**50.5**	**13,661.3**
			

Source: Internet World Stats, Datareportal, and World Bank Development Indicators

**Table 2A:** Lockdown, Partial Lockdowns, and Reopening Dates among Key Caribbean Countries

Country	Lockdown Date	Partial Lockdown Date	Reopening Date
**Bahamas**	**March 24, 2020**	**June 1, 2020**	**July 1, 2020**
**Barbados**	**March 28, 2020**	**July 1, 2020**	**July 23, 2020**
**Trinidad and Tobago**	**March 30, 2020**	**June 9, 2020**	**June 22, 2020**
**Jamaica**	**March 18, 2020**	**June 2, 2020**	**June 15, 2020**
**Guyana**	**April 3, 2020**	**July 3, 2020**	**October 12, 2020**
			

Source: Blavatnik School of Government, University of Oxford: COVID-19 Government Response Tracker

**Fig. 3A:**
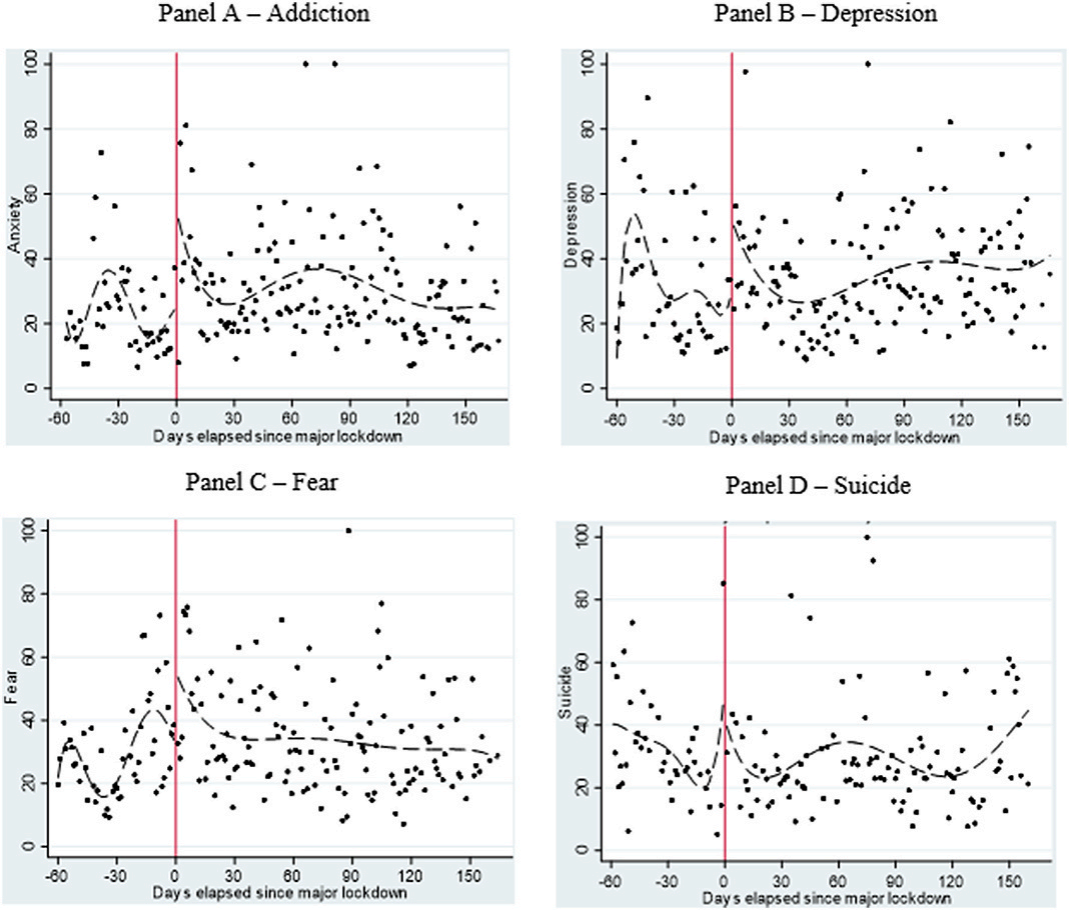
Results of RDD (2020) models based on research patterns for keywords related to mental health generated before and after implementing **Shelter in Place Orders (SIPOs)** among key Caribbean Countries (Panel A – Anxiety, Panel B – Depression, Panel C – Fear, and Panel D – Suicide). - On the vertical axis, the scaled number of searches (ranging from 0 to 100) is displayed for the days preceding (represented by negative values) and following (represented by positive values) the announcement of the Shelter-In_Place Orders (SIPOs) in 2020. The Shelter in Place Orders announcement is considered day zero. The dots on the graph represent the raw averages calculated for each day, grouped into bins of one day for five countries, weighted by the respective population size of each country.

**Fig. 4A:**
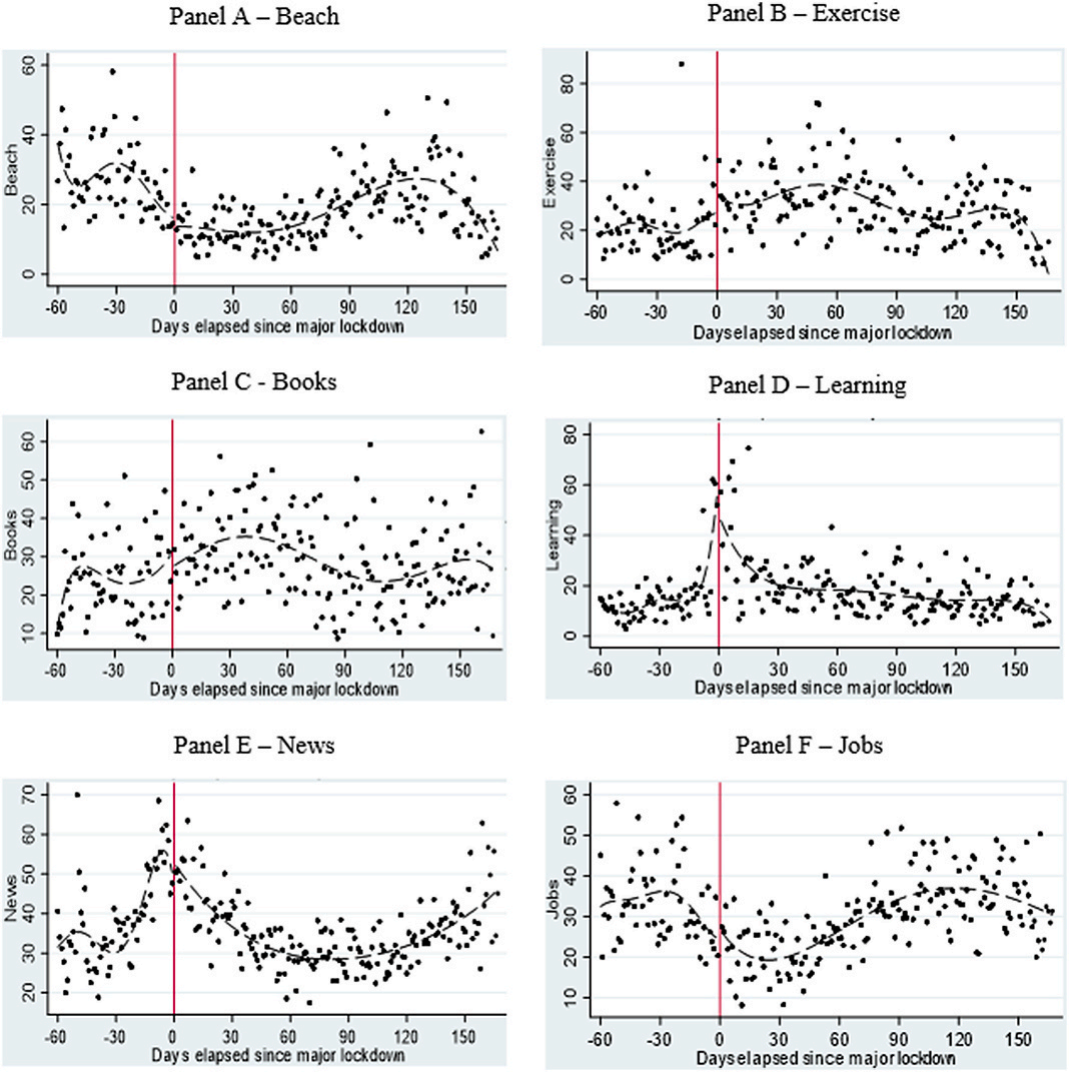
Results of RDD (2020) models based on research patterns for keywords related to positive coping strategies generated before and after implementing Shelter in Place Orders (SIPOs) among key Caribbean Countries (Panel A – Beach, Panel B – Exercise, Panel C – Books, Panel D – Learning, Panel E − News, and Panel F – Jobs). On the vertical axis, the scaled number of searches (ranging from 0 to 100) is displayed for the days preceding (represented by negative values) and following (represented by positive values) the announcement of the Shelter-In_Place Orders (SIPOs) in 2020. The Shelter in Place Orders announcement is considered day zero. The dots on the graph represent the raw averages calculated for each day, grouped into bins of one day for five countries, weighted by the respective population size of each country.

**Fig. 5A:**
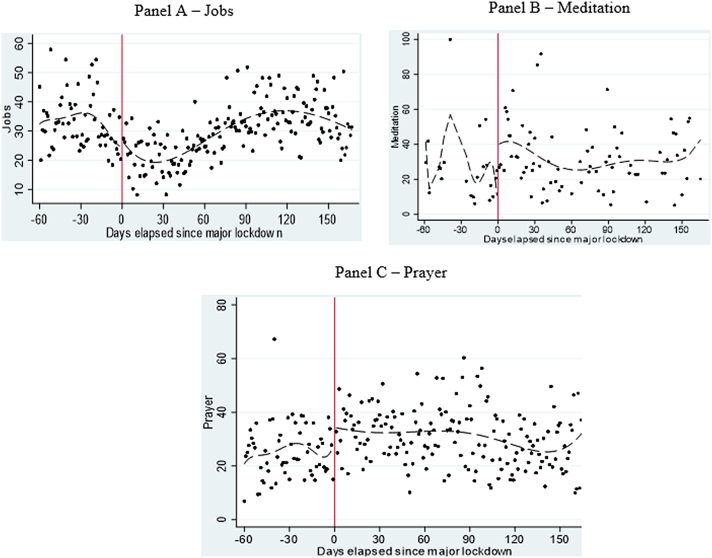
Results of RDD (2020) models based on research patterns for keywords related to religious coping strategies generated before and after implementing **Shelter in Place Orders (SIPOs)** among key Caribbean Countries (Panel A – Jobs, Panel B – Meditation, and Panel C – Prayer). - On the vertical axis, the scaled number of searches (ranging from 0 to 100) is displayed for the days preceding (represented by negative values) and following (represented by positive values) the announcement of the Shelter-In_Place Orders (SIPOs) in 2020. The Shelter in Place Orders announcement is considered day zero. The dots on the graph represent the raw averages calculated for each day, grouped into bins of one day for five countries, weighted by the respective population size of each country.

**Fig. 6A:**
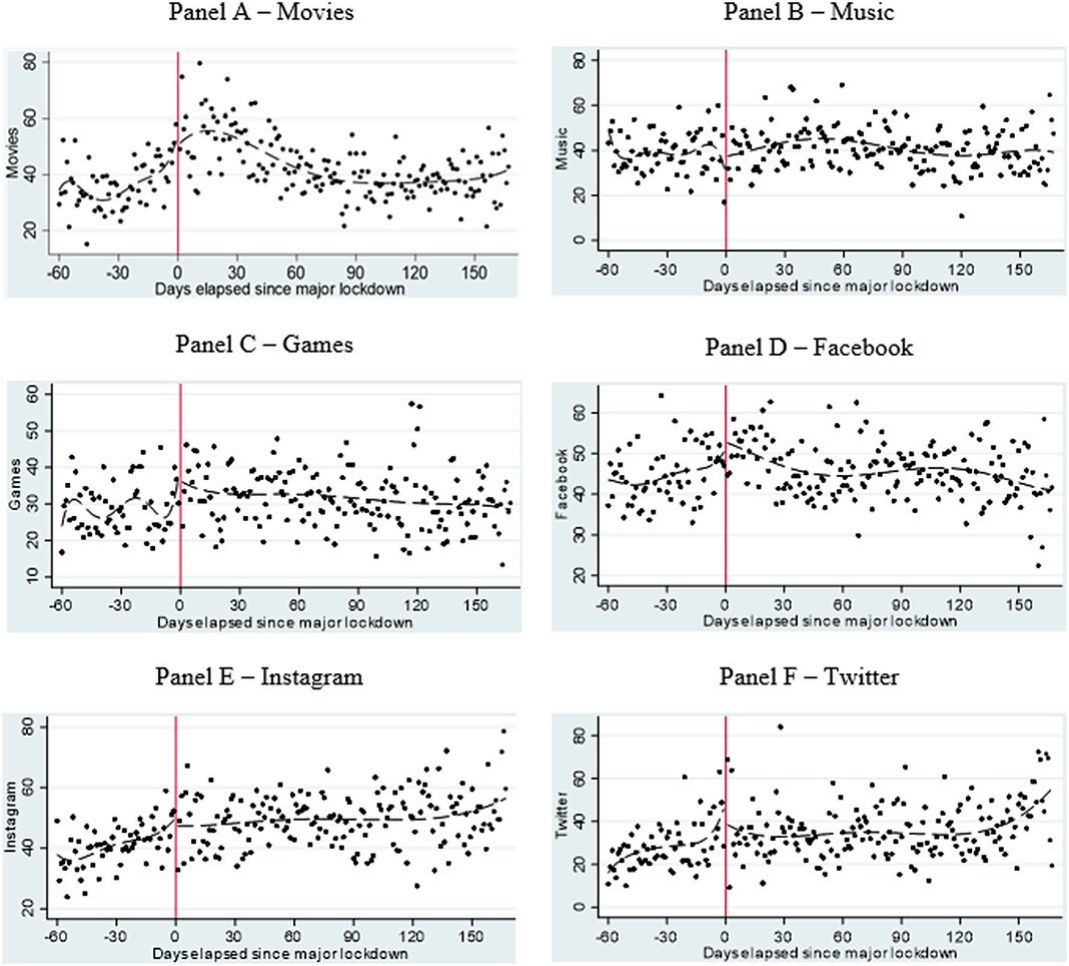
Results of RDD (2020) models based on research patterns for keywords related to distractive coping strategies generated before and after implementing **Shelter in Place Orders (SIPOs)** among key Caribbean Countries (Panel 6A – Movies, Panel 6B – Music, Panel 6C – Games, Panel 6D – Facebook, Panel 6E – Instagram and Panel 6F – Twitter). - On the vertical axis, the scaled number of searches (ranging from 0 to 100) is displayed for the days preceding (represented by negative values) and following (represented by positive values) the announcement of the Shelter-In_Place Orders (SIPOs) in 2020. TheShelter in Place Orders announcement is considered day zero. The dots on the graph represent the raw averages calculated for each day, grouped into bins of one day for five countries, weighted by the respective population size of each country.

## Rescaling process for google trends index

Brodeur, Clark [40] developed the formula to calculate the rescaled Google scores.

$$D_{i,c,2019-2020}=D_{i,c,2019}{*w}_{c,2019}\;\text{in}\;2019$$

$$D_{i,c,2019-2020}=D_{i,c,2020}{*w}_{c,2020}\;\text{in}\;2020$$

where

$$w_{c,2019}=\frac{\bar{D_{i,c,2019-2020}}}{\bar{D_{i,c,2019}}}\;\text{and}\;w_{c,2020}=\frac{\bar{D_{i,c,2019-2020}}}{\bar{D_{i,c,2020}}}$$

Where $D_{i,c,2019-2020}$ represents the rescaled Google score for each topic search in country *c* on day *i*, over the period 2019 to 2020. This is generated by multiplying daily scores for a given topic search in country *c* given by $D_{i,c,2020}$ by a weekly weight generated for each topic search by country in a given year during the survey period ($w_{c,2019}$ and $w_{c,2020}$). Relative weights are developed by finding the ratio of weekly indexed scores downloaded from Google Trends for topic searches covering the entire sample period, January 1st^,^ 2019 to September 1, 2020 $\bar{\left(D_{i,c,2019-2020}\right)}$ and calculated weekly averages for each word search. Calculated averages are found using the raw daily search data over the period January 1, 2019 and September 1, 2019 $\left(\bar{D_{i,c,2019}}\right)$ and January 1, 2020 and September 1, 2020 $\left(\bar{D_{i,c,2019}}\right)$ respectively.

## Footnotes

1 The Caribbean countries utilized for this study include Jamaica, Trinidad and Tobago, Bahamas, Barbados, and Guyana, collectively representing 78% of the total population of English-speaking CARICOM countries. Additionally, we focus our studies on the larger English-speaking Caribbean countries, where internet penetration rates remain relatively high.

2 For this paper data are gathered based on topic searches.

3 It should be noted that given the sampling methodology used to produce a representative sample dataset, results may vary each time a query is made on Google Trends 46. Google, *FAQ about Google Trends data.* 2020.

4 RSV<1 are excluded from the searches.

5 Results were also robust to variation models up to 10 days before and after.

6 The regression discontinuity design results for mental health and coping strategies based on 2019 data reflect nominal changes in search patterns over the same period in 2019 and are available upon request.
